# Mitochondrial genome from the lichenized fungus *Peltigera rufescens* (Weiss) Humb, 1793 (Ascomycota: Peltigeraceae)

**DOI:** 10.1080/23802359.2021.1944374

**Published:** 2021-07-05

**Authors:** Lidan Wang, Reyim Mamut

**Affiliations:** College of Life Science and Technology, Xinjiang University, Urumchi, China

**Keywords:** *Peltigera rufescens*, mitochondrial genome, phylogenetic analysis

## Abstract

Known colloquially as ‘dog-lichens’ or ‘pelt-lichens’, most species of *Peltigera* grow on soil and mosses. Some species contribute a significant amount of nitrogen to the environment and have been used as traditional medicines. We analyzed the complete mitochondrial genome of *P*. *rufescens*, which is a circular genome 65,199 bp in size and its CG content is 26.7%. It contains 15 protein-coding genes (PCGs), 27 transport RNAs (tRNAs), and 3 ribosomal RNAs (rRNAs). Also, the *atp*9 gene is present in the genome. We used the complete mitochondrial genome to construct a phylogenetic tree by the Bayesian method, which was consistent with the phylogenetic relationship published for *P*. *membranacea* which is closely related to *P*. *rufescens*.

*Peltigera rufescens* is a species of lichen-forming fungi, belonging to the family Peltigeraceae (Ascomycota), which is a widely distributed species around the world (Eriksson and Winka 1998). The thallus of *P*. *rufescens* is gray to brown, usually with a heavy tomentum on the upper surface (hence the name dog-lichens). The lower surface has distinctly raised veins, the apothecia is commonly dark red-brown, saddle-shaped, and on upright lobes (Miadlikowska and Francois 2000). In recent years, several studies have suggested that lichenized fungus can be used as a possible food supplement and as an easily available natural source of medicines. Studies have shown that *P*. *rufescens* extracts have antioxidant, anti-inflammatory, and antigenotoxic effects. It has an important application for the protection of human lymphocytes from the genotoxic damages induced by agricultural chemicals hazardous to people (Tanas et al. 2010; Aydin and Tuerkez 2011; Türkez et al. 2012). Therefore, *P*. *rufescens* has an important practical research significance.

The sample of *P. rufescens* was collected from Haxionggou, in Xinjiang Province (87°59.846′ N, 43°48.783′ E). This voucher specimen was deposited in the Herbarium of College of Life Science and Technology at Xinjiang University in Urumchi, China (https://mail.163.com/, for more information about this voucher please contact Reyim MAMUT, email: arman99@163.com) under the voucher number 201899269A. DNA was extracted using the NEBNext®UltraTM DNA Library Prep Kit for Illumina (NEB, USA) Kit. The sequence was performed in Novaseq PE150. The whole library was prepared by terminal repair, adding A tail, adding sequencing connector, purification, PCR amplification, and other steps. After the library construction was completed, the library was initially quantified using Qubit 2.0 and diluted to 2 ng/µL. Using readfq to capture all data for QC (Quality control). Sequence splicing used SPAdes (http://bioinf.spbau.ru/SPAdes/), then used Gapcloser and Gapfiller for scaffold gap, and finally used PrInSeS-G for sequences correction. The mitochondrial genome was annotated by GeSeq and Geneious (Kearse et al. 2012; Tillich et al. 2017), and the whole genome sequence data was deposited in GenBank under the accession number MW711788.

The total length of the mitochondrial genome of *P. rufescens* is 65,199 bp and CG content is 26.7%. The complete mitochondrial genome of *P*. *rufescens* contains 15 PCGs, 27 tRNAs, and 3 rRNAs. The genome presented here contained a conserved set of 15 PCGs (*cox*1, *cox*2, *cox*3, *nad*1, *nad*2, *nad*3, *nad*4, *nad*4L, *nad*5, *nad*6, *atp*6, *atp*8, *atp*9, *cyt*b, and *rps*3), which are arranged in the same order as in *P*. *membranacea.* In some lichen lineages, the *atp*9 gene is lost (Pogoda et al. 2018), such as *Usnea* (Lan and Huang 2020). However, the *atp*9 gene is present in *P. rufescens*, and introns invade five different genes: four PCGs (*cox*1, *cyt*b, *nad*4L, and *nad*5), one site in rRNA (rnl), we predicted a total of 11 introns I, including intron IA (6), intron IB (1), intron IC (2), and intron ID (1).

We used 11 complete mitochondrial genomes to conduct a phylogenetic analysis of *Peltigera rufescens*, using MrBayes v3.2 (Huelsenbeck and Ronquist 2001). The phylogenetic tree is divided into two clades, *Cladonia* clade and Peltigerales clade, *Opegrapha vulgata* is set as an outgroup. The phylogenetic tree indicated that *P. rufescens* is closely related to *P. membranacea* (Figure 1).

**Figure 1.**
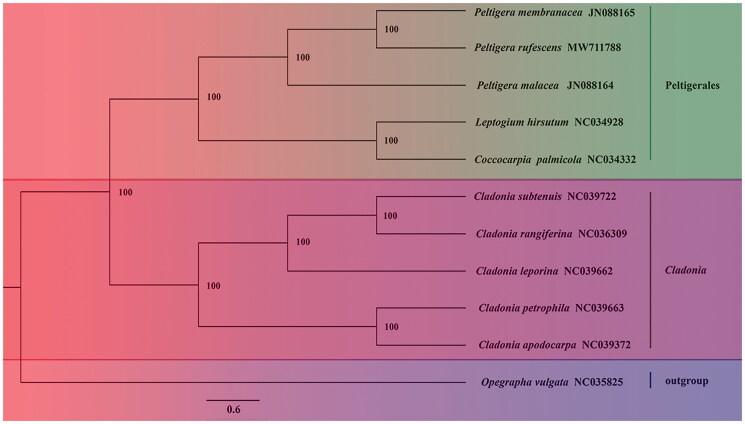
Phylogenetic analysis of *Peltigera rufescens.* A phylogenetic tree was constructed from the total length of the mitochondrial genomes of 11 species by using the Bayesian method, five species of *Cladonia* and five species of the order Peltigerales, *Opegrapha vulgata* is set as an outgroup, BI-PP = 100%.

## Data Availability

The genome sequence data that support the findings of this study are openly available in GenBank of NCBI at https://www.ncbi.nlm.nih.gov under the accession no. MW711788. The associated BioProject, SRA, and BioSample numbers are PRJNA656065, SRR14561385, and SAMN19223377 respectively.
